# Characterization of the first patient with disseminated coccidioidomycosis and autosomal dominant STAT1 deficiency

**DOI:** 10.70962/jhi.20250015

**Published:** 2025-07-29

**Authors:** Aidé Tamara Staines-Boone, Miyuki Tsumura, STAT1 Consortium, Adriana de J Rodríguez, Diana Olguín Calderón, Laura Berrón Ruiz, Jorge García Campos, Julieta Marmolejo-Bijnsdorp, María Jiménez Juárez, Carlos Sanchez Flores, Tom Le Voyer, Sara Espinosa Padilla, Jacinta Bustamante, Satoshi Okada, Lizbeth Blancas-Galicia

**Affiliations:** 1 Immunology Department, UMAE 25, IMSS, Monterrey, Mexico; 2 Hiroshima University Graduate School of Biomedical and Health Sciences, Hiroshima, Japan; 3 Infectious Disease Department, UMAE 25, Monterrey, Mexico; 4 Laboratory of Human Genetics of Infectious Diseases, Necker Branch, Necker Hospital for Sick Children, Paris, France; 5 Paris Cité University, Imagine Institute, Paris, France; 6 Study Center for Primary Immunodeficiencies, Necker Hospital for Sick Children, Assistance Publique – Hôpitaux de Paris, Paris, France; 7 St Giles Laboratory of Human Genetics of Infectious Diseases, Rockefeller Branch, Rockefeller University, New York, NY, USA; 8 Immunodeficiency Laboratory, National Institute of Pediatrics Mexico City, Mexico City, Mexico

## Abstract

A heterozygous STAT1 mutation (K410E) was identified in a child with disseminated coccidioidomycosis. This report highlights the critical role of IFN-γ–mediated immunity in controlling fungal infections and expands the known phenotypic spectrum of autosomal dominant STAT1 deficiency.

## Teaser

Autosomal dominant STAT1 deficiency is a monogenic defect that increases susceptibility to coccidioidomycosis in humans.

Signal transducer and activator of transcription 1 (STAT1) is a transcription factor that controls the cellular responses to type I, II, and III interferons (IFNs) and interleukin-27. Genetic analysis of human infectious diseases has led to the identification and characterization of four types of inborn errors of immunity (IEI) associated with STAT1: (I) autosomal recessive (AR) complete STAT1 deficiency, (II) AR partial STAT1 deficiency, (III) autosomal dominant (AD) STAT1 deficiency, and (IV) AD gain of STAT1 activity (1, 2). In AD loss-of-function (LOF) deficiencies, the disease is caused by the dominant-negative (DN) effect of mutant *STAT1* on wild-type (WT) *STAT1* and characterized by impaired GAF-dependent type II IFN immunity, with some degree of ISGF3-dependent type I IFN immunity partially preserved (1). Intracellular bacterial infections caused by disseminated *Mycobacterium bovis* Bacillus Calmette–Guérin (BCG) vaccine, nontuberculosis mycobacteria, e.g., *Mycobacterium avium*, frequently manifesting as multifocal osteomyelitis, are commonly observed in patients with AD STAT1 deficiency (1). In this study, we identified the first case of disseminated coccidioidomycosis in a patient with AD STAT1 deficiency.

**Figure 1.**
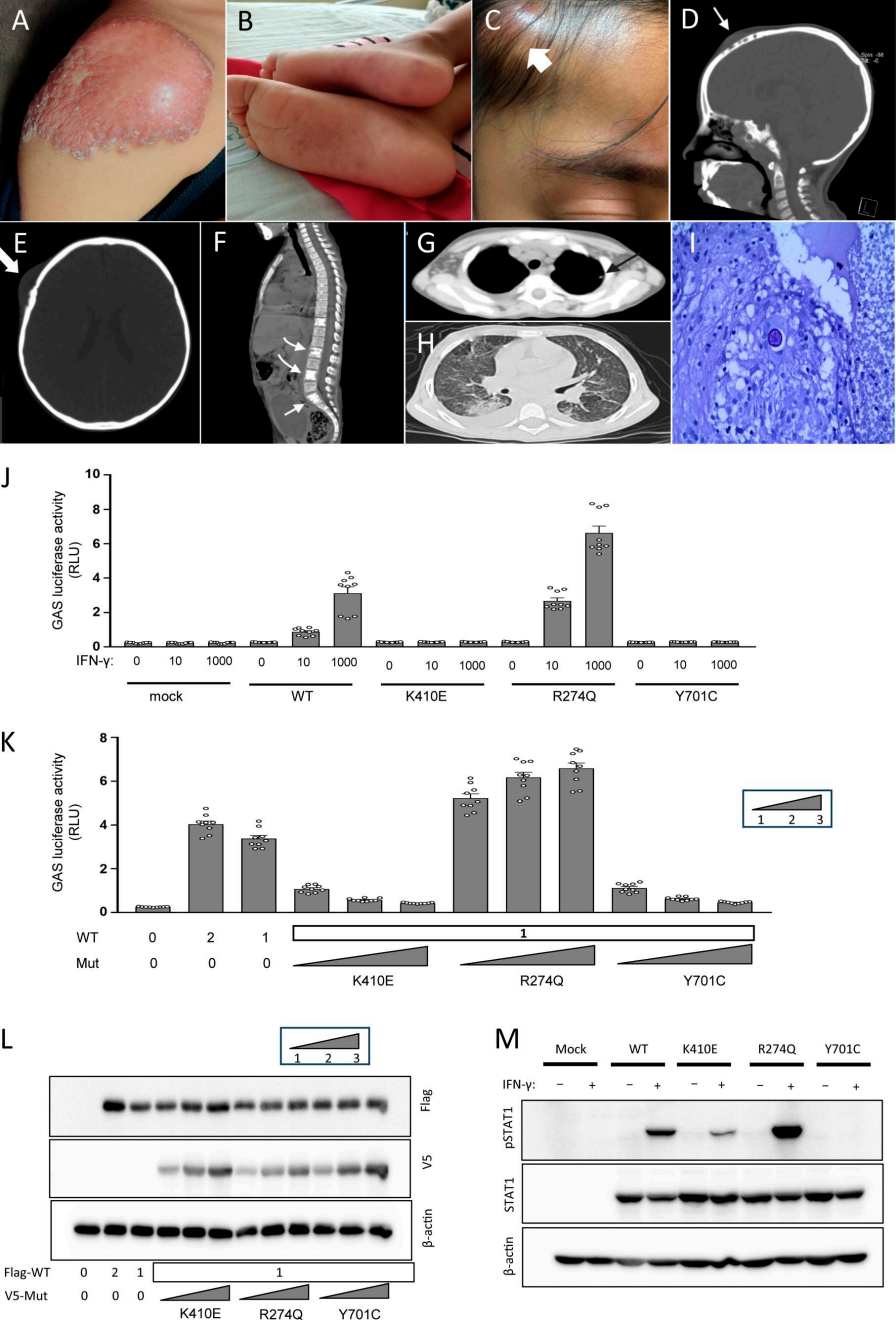
**Autosomal dominat STAT1 deficiency in a girl with coccidiodomycosis.** **(A)** Erythematous and verrucous plaque in the right deltoid region at 16 mo of age. **(B)** Dermal vasculitis of the sole associated with SARS-CoV-2 infection. **(C)** Inflammatory tumors (arrow) in the soft tissues of the right frontotemporal region. **(D)** Lytic lesions in the frontotemporal (arrow) bones overlying the soft tissue tumors revealed using CT. **(E)** Cranial CT shows lytic lesions in the temporal bone overlying the soft tissue tumors (sagittal). **(F)** Thorax CT shows lytic lesions in the L1, L2, L4, and S1 vertebrae. **(G)** Thorax CT indicates a lung nodule in the right basal lobe. **(H)** Thorax CT shows miliary pattern images. **(I)** Microscopic observation of spherule images after periodic acid–Schiff staining indicated *Coccidioides* spp. in the soft tissue specimens of the frontotemporal region. Evaluation of the variant of STAT1 K410E (J–M). **(J)** U3C cells were transfected with plasmids carrying empty vector (mock), WT, K410E, Y701C (a known LOF), or R274Q (a known gain-of-function) mutants at 2 ng/well, along with the reporter plasmids (Cignal GAS Reporter Assay Kit, QIAGEN). The total amount of the STAT1 plasmid was kept constant at 5 ng/well by supplementing with the mock vector. 24 h after transfection, the transfectants were stimulated with IFN-γ at 10 or 1,000 IU/ml for 16 h. Subsequently, the luciferase assays were performed with the Dual-Glo Luciferase Assay System (Promega). The results shown in the bar graphs represent the mean ± SEM from three independent experiments. **(K and L)** The evaluation of STAT1 variants under conditions where varying amounts of WT (1 or 2 ng) and/or mutant (1, 2, or 3 ng) STAT1 plasmids were cotransfected, with the total plasmid amount maintained at 5 ng/well using the mock vector. The expression of introduced STAT1 was evaluated by immunoblot. GAS reporter activity was measured after stimulation with 1,000 IU/ml IFN-γ. These experiments were performed to assess the effect of STAT1 mutants on WT-mediated GAS induction. The results shown in the bar graphs represent the mean ± SEM from three independent experiments. **(M)** U3C cells transiently expressing WT or mutant STAT1 were stimulated with 1,000 IU/ml of IFN-γ for 15 min and then subjected to immunoblot analysis. The following primary antibodies were used: anti-STAT1, anti-phosphorylated STAT1 at Tyr-701 (pSTAT1), and anti-β-actin. SARS-CoV-2, severe acute respiratory syndrome coronavirus 2; SEM, standard error of the mean. RLU: relative luminescence units. Source data are available for this figure: SourceData F1.

A female patient from northwest Mexico with no history of consanguinity had two healthy siblings (one brother: 15 years old, and one sister: 8 years old). She received the BCG vaccine in the right deltoid region at birth. 4 mo later, she experienced local adverse effects characterized by persistent erythematous papules and verrucous plaque (Fig. 1 A). At 18 mo of age, she was hospitalized for lobar pneumonia, which improved after a week of intravenous antibiotic treatment. At 2 years of age, the patient developed varicella without any complications. At 3 years of age, she presented with herpes zoster in the thorax. At 5 years of age, she presented SARS-CoV-2 infection despite being vaccinated, with vasculitis on the skin of her foot sole (Fig. 1 B). At 6 years 2 mo old, she was hospitalized due to persistent fever, night sweats, two inflammatory tumors (1 × 1 cm) in the soft tissues of the right frontotemporal region (Fig. 1 C), and hepatosplenomegaly. GeneXpert *Mycobacterium tuberculosis* tests of gastric juice, bronchoalveolar fluid, and bone were negative. Moreover, mycobacterial cultures of the bone and tumors from the frontotemporal region were negative. Computed tomography (CT) scans revealed lytic lesions in the temporal bone overlying the soft tissue tumors (Fig. 1, D and E), as well as the L1, L2, L4, and S1 vertebrae (Fig. 1 F), a lung nodule in the right basal lobe (Fig. 1 G), and enlarged lymph nodes in the cervical, axillary, and retroperitoneal regions. The fundus exhibited changes suggestive of granulomas in the optic nerve. Subsequent chest CT scan revealed a miliary pattern (Fig. 1 H), whereas the liver ultrasound indicated a micronodular pattern. Mycobacterial infection was suspected, but even after 4 mo of empirical treatment with antituberculosis agents, no amelioration of symptoms was observed, and the medication was discontinued. As coccidioidomycosis is endemic to the native place of the patient, it was diagnosed via the detection of serum antibodies against *Coccidioides immitis* using an enzyme-linked immunosorbent assay (IgM: 0.493 [OD: 0.150–0.200] and IgG: 2.745 [OD: 0.150–0.200]), microscopic observation of spherule images suggestive of *Coccidioides* spp. (Fig. 1 I), and isolation of *Coccidioides* spp. from the frontotemporal tumor. The patient exhibited clinical improvement after the treatment with liposomal amphotericin B (5 mg/kg/day) and fluconazole (12 mg/kg/d) for 6 wk. Subsequently, the patient continued to receive fluconazole.

The severity and recurrence of the patient’s infections prior to the age of six, in addition to her recent admission, led us to hypothesize that she may have had IEI. For this reason, we started the diagnostic approach. Laboratory results at 6 years 6 mo old were as follows: Hb 7.4 (10.5–14) g/dl, leukocytes 11.8 × 10^9^/L (5.0–14.5), neutrophils 7,000 × 109/L (1.5–8.5), lymphocytes 3,400 × 10^9^/L (2.0–8.0), platelets 135 × 10^9^/L (200–450), IgG 2,771 (923 ± 256) mg/dl, IgA 406 (124 ± 45) mg/dl, IgM 215 (65 ± 25) mg/dl, IgE 9,322 (≤90) IU/ml, CD4^+^ 1,529 (560–2,700) cells/μl, CD3^+^ 4,199 (1,200–4,100) cells/μl, CD8^+^ 2,291 (330–1,800) cells/μl, CD19^+^ 522 (220–1,300) cells/μl, and CD16/56+ 850 (48–540) cells/μl. Human immunodeficiency virus (serology) test was negative, and the dihydrorhodamine assay result for neutrophils was similar to that in the healthy control. However, whole-exome sequencing revealed a rare variant in the heterozygous state, cDNA level: NM_001384886.1(*STAT1*): c.1228A>G/WT (p.K410E). Sanger sequencing confirmed this finding and revealed that both the parents and siblings were WT/WT. The K410E variant is located within the DNA-binding domain of the STAT1 protein. It is not described in either the National Heart, Lung, and Blood Institute Exome Sequencing Project Exome Database or the Genome Aggregation Database. However, it has previously been reported in a child with tuberculosis verrucosa cutis secondary to BCG vaccination (3).

To characterize this variant, GAS luciferase reporter assay was performed to evaluate transcriptional activation by the *STAT1* mutant (Fig. 1 J). The K410E *STAT1* behaved as a LOF protein in response to stimulation with low concentrations of IFN-γ. The luciferase activity of the GAS was also measured by cotransfection with the expression vector containing the WT *STAT1* and/or the mutant *STAT1*. The mutant K410E exerted a dose-dependent DN effect on WT-*STAT1*–mediated GAS induction after IFN-γ stimulation (Fig. 1, K and L). In the western blot, the K410E variant showed a slight decrease in phosphorylation (Fig. 1 M). Taken together, these findings indicate that the K410E variant is a LOF mutation with a DN effect and is a plausible pathogenic variant causing AD STAT1 disorder. Currently, the patient is 8 years old, exhibits weight gain, stays at home, and continues treatment with fluconazole.

Here, we present the first case of disseminated coccidioidomycosis in a patient with AD STAT1 deficiency. Coccidioidomycosis has broad clinical manifestations, ranging from asymptomatic to severe pulmonary or disseminated cases, possibly leading to death in severe cases (4). Coccidioidomycosis is an endemic infection in Mexico and other regions of the world (4). Here, the reported case indicates the influence of geographical zone on the infectious susceptibility of patients with AD STAT1 deficiency. This IEI is a monogenic cause of coccidioidomycosis in addition to those previously reported (*STAT1* GOF, *IL12RB1*, *IFNGR1*, *GATA2*, *STAT3* DN, and *CTPS1* deficiencies) (5). Outcomes of patients with coccidioidomycosis depend on the cell-mediated immunity; however, the precise mechanisms of this response remain unclear (4). AD gain of STAT1 activity predisposes patients to invasive fungal infections, including coccidioidomycosis (5). Here, the patient demonstrated an infectious overlap between AD gain in STAT1 activity and AD STAT1 deficiency (1). Therefore, further investigations are necessary to elucidate the immune responses to fungal infections associated with STAT1. This report highlights the need to test patients with AD STAT1 deficiency for coccidioidomycosis, especially those living in endemic regions of this invasive fungal infection.

## Supplementary Material

Table S1 lists the STAT1 Consortium members and affiliations.

SourceData F1 is the source file for Fig. 1.

## Data Availability

All data and materials can be obtained by contacting the corresponding author.
