# Supplementary material for: Characterization of the first patient with disseminated coccidioidomycosis and autosomal dominant STAT1 deficiency
Source: J Hum Immun. 2025 Jul 29;1(3):e20250015. doi: 10.70962/jhi.20250015 (PMC12829754; doi:10.70962/jhi.20250015)
Supplement: Table S1 — lists the STAT1 Consortium members and affiliations. [file jhi_20250015_tables1.pdf]

STAT1 CONSORTIUM AUTHORS

Adriana de J Rodríguez[1**], Diana Olguín Calderón [2,3], Laura Berrón Ruiz[4], Jorge García Campos[1], Julieta Marmolejo-Bijnsdorp[5], María Jiménez Juárez[4], Carlos Sanchez Flores[4], Tom Le Voyer[2,3], Sara Espinosa Padilla[4]

CONSORTIUM AFFILIATION

1. Infectious Disease Department, UMAE 25, Monterrey, Nuevo Leon, Mexico
2. Laboratory of Human Genetics of Infectious Diseases, Necker Branch, Necker Hospital for Sick Children, Paris, France.
3. Paris Cité University, Imagine Institute, Paris, France.
4. Immunodeficiency Laboratory, National Institute of Pediatrics Mexico City, Mexico.
5. Immunology Department, UMAE 25, IMSS, Monterrey, Nuevo Leon, Mexico.
