# Supplementary material for: Characterization of the first patient with disseminated coccidioidomycosis and autosomal dominant STAT1 deficiency
Source: J Hum Immun. 2025 Jul 29;1(3):e20250015. doi: 10.70962/jhi.20250015 (PMC12829754; doi:10.70962/jhi.20250015)
Supplement: SourceData F1 — is the source file for Fig. 1. [file jhi_20250015_sourcedataf1.pdf]

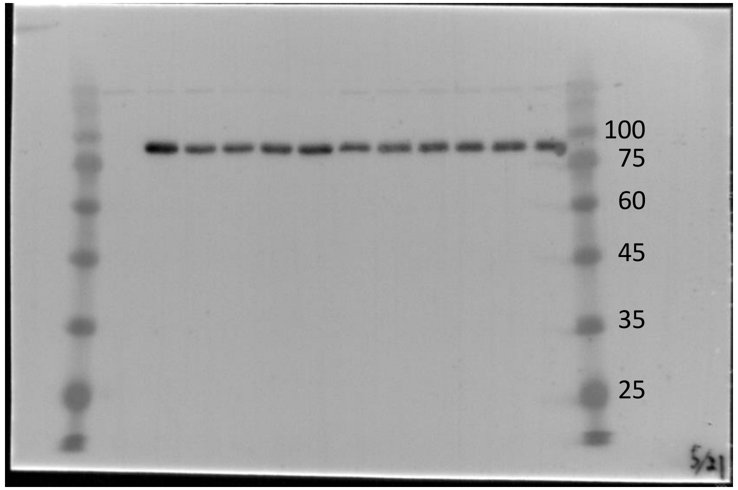

Flag-WT 0 2 1 1

V5-Mut 0 0 0

K410E R274Q Y701C

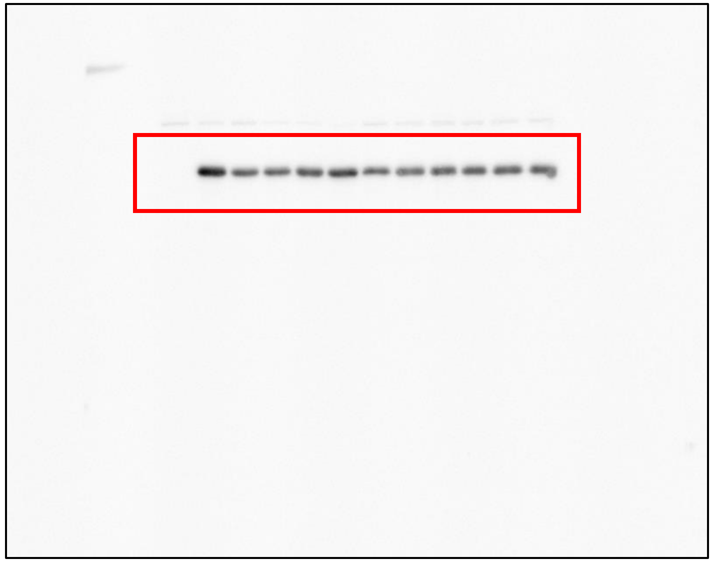

Flag

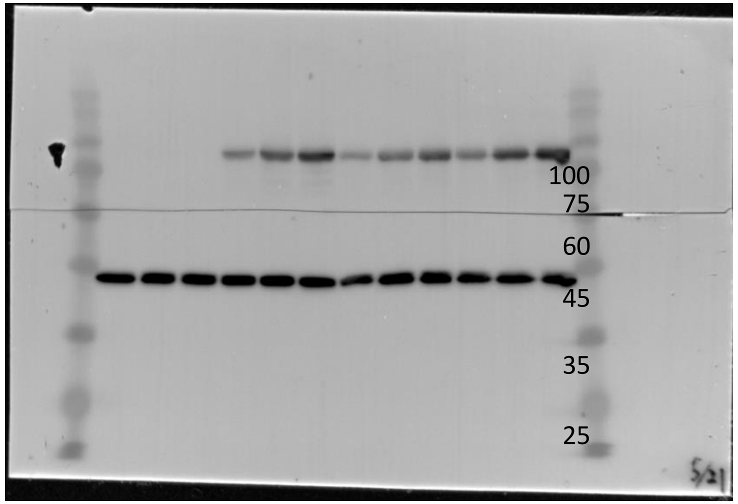

Flag-WT 0 2 1 1

V5-Mut 0 0 0

K410E R274Q Y701C

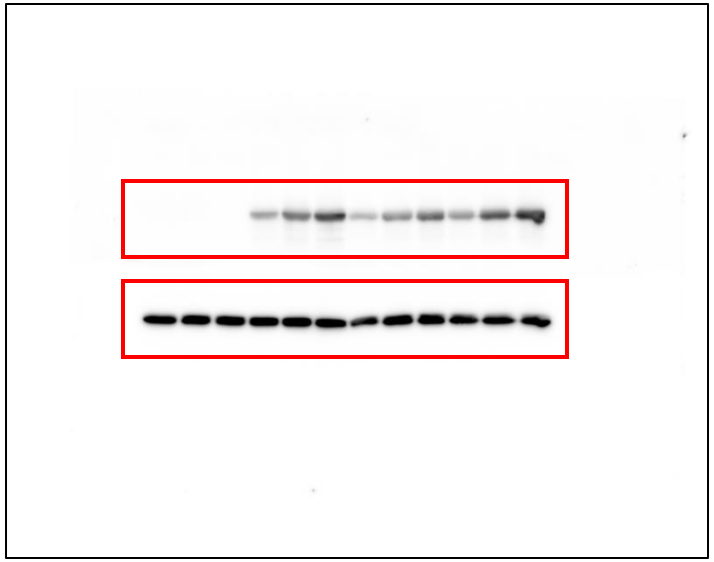

V5

β-actin

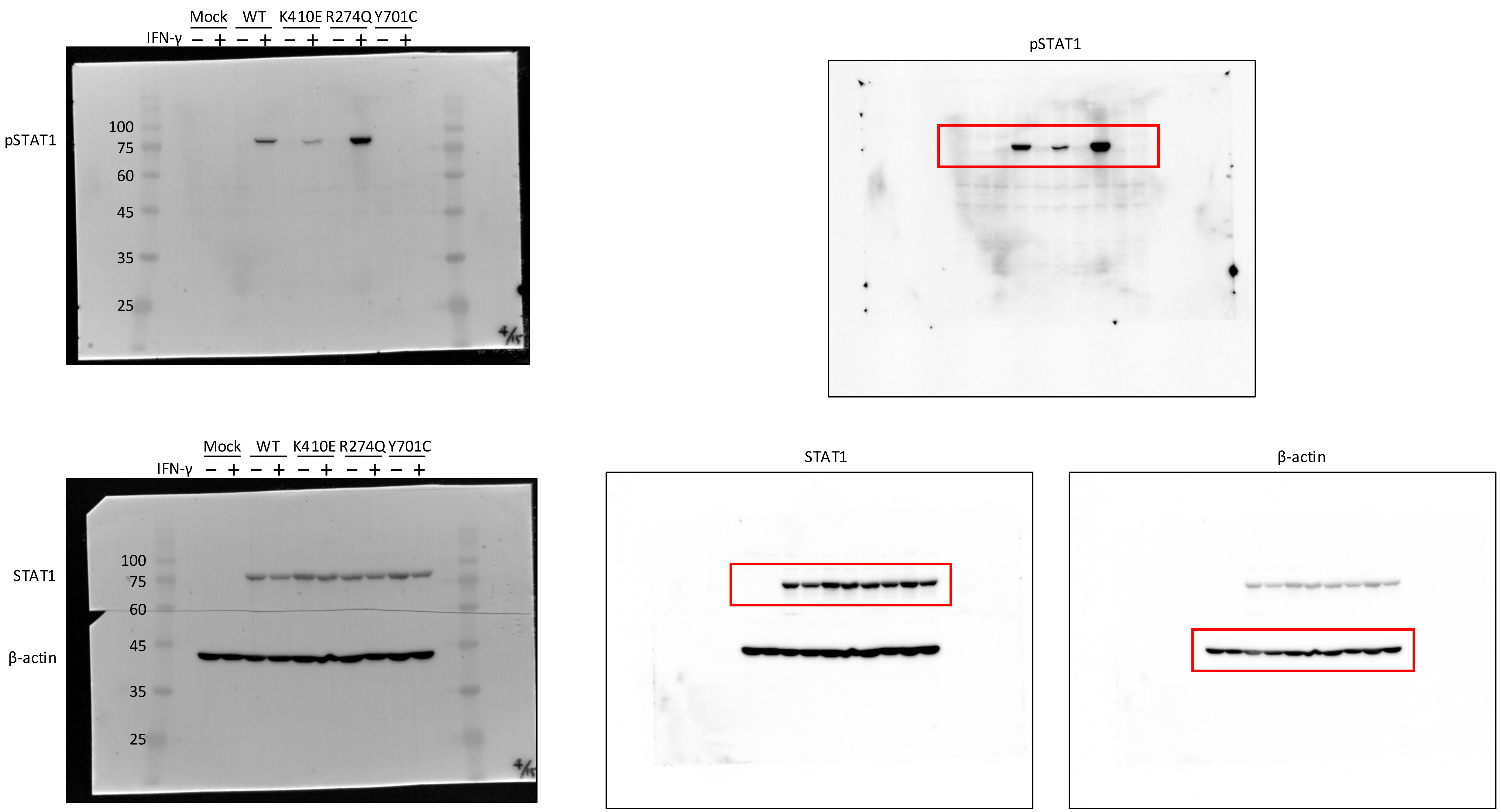
Mock
WT
K410E
R274Q
Y701C
IFN-γ − + − + − + − + − +
pSTAT1
100
75
60
45
35
25
pSTAT1
Mock
WT
K410E
R274Q
Y701C
IFN-γ − + − + − + − + − +
STAT1
β-actin
100
75
60
45
35
25
STAT1
β-actin
